# Effects of inhaled nitric oxide (iNO) in pulmonary hypertension secondary to arteriovenous malformations: a retrospective cohort study from the European iNO registry

**DOI:** 10.1007/s00431-022-04602-9

**Published:** 2022-09-06

**Authors:** Aravanan Anbu Chakkarapani, Samir Gupta, Asma Jamil, Santosh Kumar Yadav, Nim Subhedar, Helmut D. Hummler

**Affiliations:** 1 Division of Neonatology, Sidra Medicine, Doha, Qatar; 2 Weill Cornell Medicine-Qatar, Doha, Qatar; 3 Durham University, Durham, UK; 4 Research Department, Sidra Medicine, Doha, Qatar; 5 Johns Hopkins University School of Medicine, Baltimore, MD USA; 6 Liverpool Women’s Hospital, Liverpool, UK; 7 University of Tuebingen, Tuebingen, DE Germany

**Keywords:** Nitric oxide, Pulmonary hypertension, Arteriovenous malformations, Oxygenation index, Hemodynamics, Neonates

## Abstract

This study aims to assess the effects of inhaled nitric oxide (iNO) on oxygenation in the management of pulmonary hypertension (PH) secondary to arteriovenous malformations (AVMs) in neonates. This is a matched retrospective cohort study from January 1, 2013, to December 31, 2017. The European inhaled nitric oxide registry from 43 neonatal and pediatric ICUs in 13 countries across Europe was used to extract data. The target population was neonates treated with iNO for the management of PH. The cases (PH secondary to AVMs treated with iNO) were matched (1:4 ratio) to controls (PH without AVMs treated with iNO). The main outcome measure was the absolute change of oxygenation index (OI) from baseline to 60 min after starting iNO in cases and controls. The primary outcome of our study was that the mean absolute change in OI from baseline to after 60 min was higher among cases 10.7 (14), than in controls 6 (22.5), and was not statistically different between the groups. The secondary outcome variable — death before discharge — was found to be significantly higher in cases (55%) than in controls (8%). All the other variables for secondary outcome measures remained statistically insignificant.

*   Conclusion*: Infants with PH secondary to AVMs treated with iNO did not respond differently compared to those presented with PH without AVMs treated with iNO. Right ventricular dysfunction on echocardiography was higher in cases than controls (cases: 66.7% and controls: 28.6%) but was not statistically significant.

**Table:** 

**What is Known:** *• Arterioenous malformation (AVM) is a well-known cause of persistent pulmonary hypertension in newborns. Inhaled nitric oxide (iNO) is most commonly used as first-line therapy for pulmonary hypertension in newborns.* • *Around 40–50% of vein of Galen malformations (VOGMs) are found to have congestive heart failure in the neonatal period.*	
**What is New:** *• Neonates may present with an isolated PH of the newborn as the main feature of the VOGMs. A large proportion of cases with AVMs have been associated with right ventricular cardiac dysfunction. * •* Results from one of the largest database registries in the world for iNO have been used to answer our research question.*

**What is Known:**

*• Arterioenous malformation (AVM) is a well-known cause of persistent pulmonary hypertension in newborns. Inhaled nitric oxide (iNO) is most commonly used as first-line therapy for pulmonary hypertension in newborns.*

• *Around 40–50% of vein of Galen malformations (VOGMs) are found to have congestive heart failure in the neonatal period.*

**What is New:**

*• Neonates may present with an isolated PH of the newborn as the main feature of the VOGMs. A large proportion of cases with AVMs have been associated with right ventricular cardiac dysfunction. *

•* Results from one of the largest database registries in the world for iNO have been used to answer our research question.*

## Introduction

### Background

Arteriovenous malformations (AVMs) are known to be congenital disorders of the blood vessels showcasing an abnormal and complex web of arteries and veins tangled together and connected by fistulas. Arteriovenous malformations are a well-known cause of persistent pulmonary hypertension of the newborn (PPHN) [1, 2]. A recent nationwide study from Japan reported that different types of pediatric intracranial arteriovenous (AV) shunts occur, such as brain arteriovenous malformations (BAVMs), pial AV fistulas (PAVFs), the vein of Galen malformations (VOGMs), and the Dural AV fistulas (DAVFs). Out of these, those commonly seen in neonates and infants are VOGMs, DAVFs, and PAVFs whereas BAVMs are very rare to be found [3].

Approximately 40–50% of VOGMs present in the neonatal period are accompanied by congestive heart failure [4, 5], and occasionally, neonates present with isolated PPHN as the main feature of the VOGMs later develop cardiac failure [1]. Large VOGMs are challenging to manage due to chronic and excessive pulmonary blood flow in utero and postnatally, resulting in right ventricular overload and failure. Only a few cases of VOGMs, secondary to pulmonary artery hypertension, are complicated by the “steal” phenomenon where a large intracerebral shunt causes hypoperfusion of the normal brain tissue thus exacerbating neurological injury. This complex pathophysiology also fosters a systemic hypoperfusion which may present as renal and hepatic dysfunction [6]. Mortality due to vein of Galen malformations (VOGMs) during the neonatal period is high, while those who survive have low rates of usual neurodevelopment [7]. Early intervention may be contraindicated by the risk of heart failure causing end-stage organ dysfunction [8]. Post-mortem histopathological findings in severe heart failure patients have been associated with marked bilateral ventricular hypertrophy and significant muscular thickening of intra-alveolar arterioles [9–12].

Inhaled nitric oxide (iNO) is most commonly used as first-line therapy for the PH of the newborn and hypoxemic respiratory failure. It has been routinely used for the treatment of PH secondary to AVMs and increased PVR. However, its use in infants with increased pulmonary blood flow (PBF) remains questionable as it can worsen the symptoms of congestive heart failure (CHF). Jerwood and Stokes reported a case of PH, where a massive pulmonary AVM was found to be the underlying cause of reassessment [13]. Hendson et al. reported using iNO to decrease pulmonary pressures in the case of cerebral AVMs. The initial response in this patient was short-lasting, and the infant’s tricuspid regurgitation gradient reverted to supra systemic levels within a short period [11]. Giesinger et al. described the use of iNO in two cases of VOGM presented with pulmonary hypertension and described the better management of these cases with the help of carefully guided bedside targeted neonatal echocardiography (TnECHO) [14].

### Rationale

Overall, we found very few case reports with insufficient data regarding the usefulness of iNO for the management of PH secondary to AVMs in the literature. It raises the question of whether iNO has any clinical effect in managing pulmonary hypertension secondary to AVMs. We have drawn on the data from the European inhaled nitric oxide (iNO) registry, a voluntary database where clinicians report clinical data on neonatal and pediatric patients receiving iNO for the management of PH.

### Objective

This study aims determine whether iNO has any clinical effect in managing PH in patients secondary to AVMs. We hypothesize that infants who receive treatment with iNO (cases) for PH secondary to AVMs do not respond (oxygenation and gas exchange) differently compared to infants with PH who receive treatment with iNO without underlying AVMs (controls).

## Methods

### Study design and setting

This is a retrospective matched cohort study from January 1, 2013, to December 31, 2017. The European iNO registry (https://www.medscinet.net/ino/) has been collecting up-to-date information about neonates who are treated with iNO from 43 neonatal and pediatric intensive care units in 13 countries across Europe. Data in the iNO registry were coded and de-identified.

### Eligibility criteria for participants

Infants were identified as cases if they were treated for PH with iNO secondary to AVMs and did not have congenital heart disease other than persistent ductus arteriosus. Infants admitted for other causes of PH and required treatment with iNO without underlying AVMs were the controls.

### Source and methods of participant selection

Study candidates were selected from the European iNO registry. In this study, the clinical evidence of labile hypoxemia and pre-post ductal saturation difference of more than 10% clinically led to the diagnosis of PH and was confirmed by echocardiography.

### Data source measurement

OI was measured by mean airway pressure ((MAP) in cmH_2_O × FiO_2_) × 100 ÷ PaO_2_.

### Matching criteria

The cases and controls were matched to minimize confounding factors, selection bias, and for efficient analysis. Neonates in the control group (PH due to respiratory distress syndrome, meconium aspiration syndrome, sepsis, congenital pneumonia, congenital diaphragmatic hernia, pulmonary hypoplasia, chronic lung disease, and perinatal hypoxic ischemia receiving treatment with iNO without underlying AVMs) were matched to case group (PH secondary to AVMs receiving treatment with iNO). The matching was done using two or more of the following criteria: gestational age ± 1 week, birthweight ± 100 g, and the year of admission. Matching for gestational age ± 1 week or birthweight ± 100 g was a mandatory requirement.

### Matching ratio

The cases (PH secondary to AVMs receiving treatment with iNO) were extremely rare to find; hence, we choose to select four matched controls (PH secondary to other causes (except AVMs) receiving treatment with iNO) for each case. To minimize recruitment bias, a database coordinator who was not involved in the study identified the matched controls.

### Primary outcome

The primary outcome measure of the study was the absolute change of OI from baseline to after 60 min of starting iNO between the two groups: cases and control.

### Secondary outcome

We have included the following variables: fractional-inspired oxygen concentration (FiO2), oxygen saturation (SPO2), partial pressure of oxygen (PaO2), P/F ratio (PaO2/FiO2), mean airway pressure, use of invasive high-frequency oscillation, use of surfactant, mean systemic arterial pressure, heart rate, use of inotropes and or vasotropes, use of intravenous vasodilators, use of Extra Corporeal Membrane Oxygenation (ECMO), and death before discharge.

### Statistical methods

The data was assessed for normality distribution using Kolmogorov–Smirnov. Due to the presence of violations of the assumptions of the symmetric distribution of data, we used nonparametric statistical tests for the statistical analysis. Mann–Whitney *U*-test was used for between groups (cases and controls) analysis. However, Wilcoxon Signed Rank test was used for pairwise analysis within groups. Categorical variables were analyzed using the chi-square test, and the data were presented in percentages. We considered the two-sided *P* value of less than 0.05 to be statistically significant. All the data were analyzed in SPSS Version 22 (IBM Corporation, Armonk, NY, USA).

## Results

**Fig. 1:**
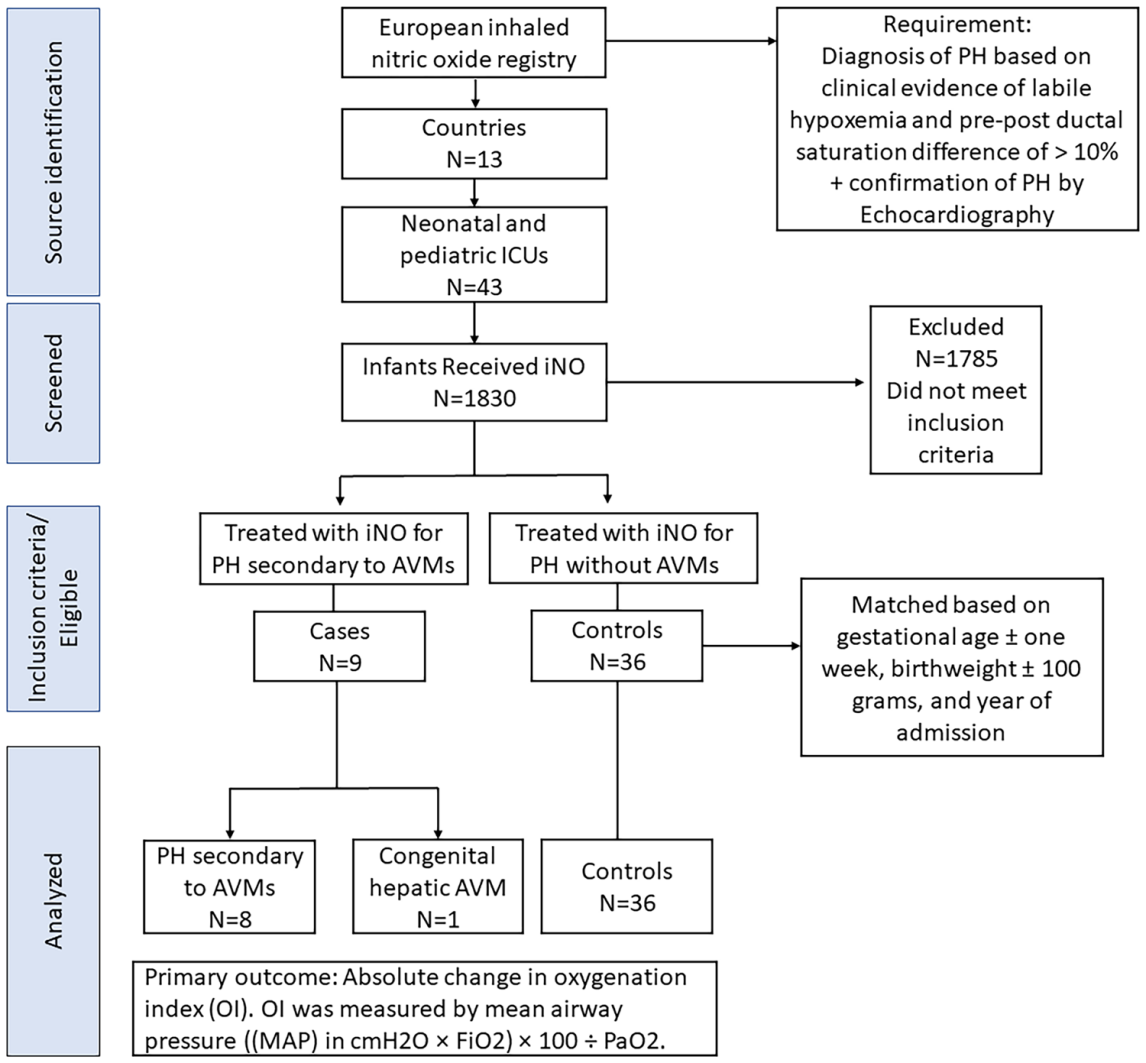
Flow diagram illustrating infants through the study

The European inhaled nitric oxide registry from 43 neonatal and pediatric ICUs in 13 countries across Europe was used to extract data. A total of 45 infants (9 cases and 36 controls) were identified from 1830 infants who received iNO during the study period. There were 8 cases of PH secondary to the vein of Galen AVMs, and one case with congenital hepatic AVM that received treatment with iNO as shown in Fig. 1.

Demographic data including gestational age at birth, birth weight, and gender are summarized in Table 1; other variables are presented in the tables with appropriate measures (mean, standard deviation, median, interquartile range). There was no difference in the baseline demographics (gestational age at birth, birth weight, and gender) between cases and controls (Table 1). Mean (SD) maintenance iNO dose (ppm) was significantly lower among cases (11.8) compared to the controls (17.6); *P* = 0.04 (Table 1). The echocardiographic findings are shown in Table 1 for both cases and controls. The echocardiographic evidence of right ventricular dysfunction was observed to be higher in cases (66.7%) as compared to controls (28.6%) but not statistically significant (*P* = 0.05).

**Table 1:** Biophysical and demographic characteristics of cases and controls, dose, and duration of inhaled nitric oxide between cases and controls, and echocardiographic variables between cases and controls

**Measures**	**Cases** Mean (SD)	**Controls** Mean (SD)	***P*-value**
GA at birth (in weeks)	38.6 (3.4)	38.3 (3.5)	0.74^*^
Birth weight (Kg)	3.4 (1.1)	3.53 (1.0)	0.74^*^
Male/female	7/9	25/36	0.30^**^
iNO starting dose (ppm)	19.1(10)	16.3 (5.3)	0.67^**^
iNO maintenance dose (ppm)	11.8 (6)	17.6 (6.5)	0.04^**^
iNO maximum dose (ppm)	21.6 (8)	22.3 (7.8)	0.69^**^
iNO final dose (ppm)	6.3 (7.9)	4.2 (6.6)	0.34^**^
Duration of iNO treatment(days)	2.1 (2.3)	2.9 (3.7)	0.78^**^
Evidence of pulmonary hypertension (%)	100 (8/8)	75 (15/20)	0.16^*^
Evidence of right to left shunt (%)	87.5 (7/8)	66.7 (14/21)	0.22^*^
Evidence of left ventricular dysfunction (%)	22 (2/9)	25 (5/20)	0.36^*^
Evidence of right ventricular dysfunction (%)	66.7 (6/9)	28.6 (6/21)	0.05^*^

^*^P < 0.05 was considered significant by the Mann–Whitney *U* test; ^**^P < 0.05 was considered significant by the chi-square test

The primary outcome (Table 2) of our study was, the mean (SD) absolute change in OI from baseline to after 60 min among cases was higher than control (cases: 10.7 (14), controls: 6 (22.5)) and was not statistically significant (*P* > 0.05).

**Table 2:** Primary outcome variables between cases and controls

**Primary outcome measures**	**Cases** Mean (SD)	**Controls** Mean (SD)	***P*-value**
Oxygenation index (OI) baseline (before iNO)	27.5 (2.2)	34 (2.7)	0.59
OI after 60 min of iNO	16.8 (11)	28.5 (31)	0.67
Absolute change in OI between baseline (before iNO) and after 60 min of iNO	10.7 (14)	6 (22.5)	0.32

*P* < 0.05 was considered significant by the Mann–Whitney

*U* test, *OI* oxygenation index, *iNO* inhaled nitric oxide

Among the secondary outcome variables, death before discharge was found to be significantly higher in cases (55%) than in controls (8%) (*P* = 0.004), while all the other variables for secondary outcome measures remained statistically insignificant as shown in Table 3.

**Table 3:** Secondary outcome variables between cases and controls

**Secondary outcome measures**	**Cases (*n* = 9)**	**Controls (*n* = 36)**	***P*-value**
FiO_2_ Mean (SD)	0.79 (0.21)	0.75 (0.26)	0.87^*^
SPO_2_ Pre ductal (%) Mean(SD)	92.3 (6.5)	89.7 (11.7)	0.83^*^
Arterial PaO_2_ (mmHg) Mean(SD)	85.9 (47.7)	80 (102)	0.08^*^
P/F ratio (PaO_2_/FiO_2_) Mean(SD)	112.3 (58)	121.6 (151)	0.230^*^
Mean airway pressure (in cmH_2_O) Mean(SD)	14.1 (5.4)	13.8 (4.7)	0.840^*^
Use of invasive (high-frequency oscillation) (%)	33.3 (3/9)	27.8 (10/36)	0.292^**^
Use of surfactant (%)	22 (2/9)	47 (17/36)	0.127^**^
Mean systemic arterial pressure (mmHg) Mean(SD	45.8 (13.5)	52.7 (9.8)	0.22^*^
Heart rate (beats/min) Mean(SD)	128 (47) 18	148 (31)	0.29^*^
Use of inotropes and or vasotropes (%)	66.7 (6/9)	58.3 (21/36)	0.273^**^
Use of intravenous vasodilators (%)	33.3 (3/9)	16.7 (6/36)	0.185^**^
Use of ECMO (%)	0 (0/9)	11.1 (4/36)	0.396^**^
Death before discharge (%)	55.6 (5/9)	8.3 (3/36)	0.004^**^

^*^P < 0.05 was considered significant by the Mann–Whitney; ^**^P < 0.05 was considered significant by the chi-square test

*U* test, *OI* oxygenation index, *iNO* inhaled nitric oxide, *FiO*_*2*_ Fractional inspired oxygen concentration, *SPO*_*2*_ oxygen saturation, *PaO*_*2*_ partial pressure of oxygen, *ECMO* extra corporeal membrane oxygenation

## Discussion

This is the first study to evaluate the effects of iNO on gas exchange and hemodynamics in the management of PH secondary to AVMs. We relied on one of the largest database registries in the world for iNO use in neonatal intensive care units in order to conduct this research. We observed that the absolute change in oxygenation index and hemodynamic parameters for infants with PH secondary to AVMs treated with iNO was similar to infants with other causes of PH requiring treatment with iNO without underlying AVMs. Interestingly, we found that echocardiography evidence of right ventricular dysfunction and mortality before discharge was more substantial among cases as compared to controls.

The AVMs may occur alongside PH due to considerable increase in pulmonary blood flow that overlays on the normal neonatal pulmonary venous drainage. Literature suggests that the diagnosis of PH of the newborn should be considered in cases where there is failure to respond to standard treatment. Additionally, a thorough clinical examination is critical to establish a differential diagnosis together with bedside tools such as head and abdominal ultrasound to help detect arteriovenous fistula of the vein of Galen or of the liver. Rarely do we need to use advanced imaging such as a computed tomographic angiogram or MRI angiogram of the brain, heart, lung, liver, or the great vessels to diagnose AVMs. The majority (8) of our cases consisted of PH secondary to vein of Galen AVMs while only one displayed congenital hepatic AVM, and all received treatment with iNO.

As in our study, Giesinger et al. and Dahdah NS et al. also reported that the majority of AVMs of the vein of Galen during early stages of life consisted of significant PH that prompted the use of iNO [10, 14]. On the other hand, Agha HM et al. managed the treatment of congenital hepatic AVM associated with severe PH and cardiac failure in a full-term female newborn without iNO [15]. They found spontaneous regression in the size of the feeder vessel as well as in the size of the vascular bed of the congenital hepatic AVM with the help of conservative use of oral heart failure therapy using diuretic agents and captopril. They observed a decrease in the congestion and diameter of the affected vessels. Although we do not have any pulmonary AVMs in our study population, Jerwood DC et al. reported a case of pulmonary AVM in which right-to-left shunting still occurred regardless of the therapy used [13]. Upon reassessment, substantial pulmonary AVM was found, and surgery was performed during the neonatal period, which turned out to be successful. Based on the literature findings, one may speculate that none of our study cases required iNO to manage secondary PH due to the AVMs. However, in cases of neonatal PPHN, it may be challenging to prospectively differentiate the PH with and without AVMs so early after birth.

In our study, echocardiographic evidence of right ventricular pressure is obtained by measuring the tricuspid regurgitation gradient before the initiation of iNO. The European iNO registry captured data regarding baseline echocardiography before starting iNO and follow-up echocardiography after 60 min of iNO. Hendson L et al. reported that a good initial response to iNO was available only for a few moments before the tricuspid regurgitation gradient increased back to supra systemic levels in babies with vein of Galen malformation [11]. Once the AVMs had been embolized, they observed a continued reduction in the tricuspid regurgitation gradient. Recently, Giesinger et al. reported two cases of VOGM management with iNO [14]. In this study, they used targeted neonatal echocardiography findings (pattern and direction of shunt across the patent ductus arteriosus, tricuspid annular plane systolic excursion (TAPSE), right ventricular and left ventricular output in ml/kg/min, and ratio, an ejection fraction of left ventricle) and suggested medical management based on pathophysiological phenotype.

Heuchan A.M et al. and Patel N et al. reported the use of superior vena cava (SVC) flow measurements to understand the hemodynamic changes in VOGM [16, 17]. Bicêtre scoring system was used for the clinical management and the prognostic aspects of VOGM in neonates [18]. Unfortunately, in our study, we do not have any data regarding SVC flow and clinical scoring from the European iNO registry for the management and prognosis of babies with VOGM.

There might be multiple reasons why our study could not find any difference to iNO in AVM-associated PH between cases and controls. Our study sample is very small. Most of the cases in our study population are referrals to a tertiary care center. The time of onset of AVMs in fetal life is unclear. The presumed first theory is that if the AVM occurs in the early part of fetal life, it will increase pulmonary vascular resistance (PVR) with remodeling structural changes in the pulmonary blood vessels due to excessive pulmonary blood flow over a period of time. The second theory is that if the AVM occurs in the later part of life, it will increase PVR without remodeling structural changes in the blood vessels. It is believed that if the PH due to AVMs develops in the early part of development, pulmonary vascular remodeling responds better to iNO compared to PH due to AVMs developing in the later part of infancy. The pathophysiology of the response to iNO during the management of AVM-associated PH is functionally unpredictable. The echocardiography at bedside is a valuable tool in understanding the pathophysiology of AVM-associated PH and the altering course of management with available treatment measures based on timely response to iNO in these conditions.

This study’s strength lies in the meticulous data collection from one of the largest iNO registries in the world, matched controls, and a design that evaluated the various outcomes of infants with PH secondary to AVMs. Furthermore, this is the most extensive collection of cases of PPHN secondary to AVMs that are treated with iNO.

A number of limitations need to be acknowledged. Firstly, in an ideal setting, the control group would have consisted of those infants that have had PH secondary to AVMs but did not receive iNO. However, this approach was beyond the scope of our study design. Secondly, the timing of the diagnosis of AVMs in cases was not defined a priori in our study. Thus, the selection of infants for further investigation and treatment could have been subjected to the bias of the individual institutions’ caretakers. Thirdly, echocardiographic evidence of PH has not been followed systematically with timed serial echocardiography before and after the initiation of iNO in both groups as per the international point of care echocardiographic guidelines [19]. Hence, it is challenging to objectively define the improvement or deterioration of PH in infants secondary to AVMs.

Furthermore, there is no clear data available with regard to who has performed the echocardiography in our study population as the neonatologist or pediatric intensivist performing echocardiography is increasingly utilized compared to pediatric cardiologist performing echocardiography in European NICUs. Fourth, our findings indicate a significant increase in the incidence of mortality before discharge in infants with PH secondary to AVM. Such an observation could be due to selection bias or could indicate the worsening of PH and right ventricular dysfunction in infants secondary to AVM after initiation of iNO. Fifth, the European iNO registry did not capture detailed data regarding the management of refractory pulmonary hypertension managements on these babies, which includes inhalational pulmonary vasodilators, etc. [20] (in our study, as mentioned in Table 3, we have the data regarding % of the use of intravenous vasodilators and ECMO therapy in babies with refractory PH). In addition, vasoactive-inotropic score (VIS) is the validated score to measure the severity of illness and associated with outcome in critically ill babies. Unfortunately, these data were not available for our study participants from the European iNO registry’s database [21]. Lastly, the control group was matched for gestational age, birth weight, and year of admission. However, we could not match on the severity of illness (SNAP-II and SNAPPE-II) and coexisting morbidities at the time of NICU admission due to unavailability of data or difficulty in extracting the data from the European iNO registry [22].

The results of this retrospective cohort study are not generalizable for all neonates due to the limitations of the study as mentioned above. However, this study could serve as a foundation for future prospective, well-designed research to address whether the use of iNO for the treatment of PH improves oxygenation and hemodynamics in infants with hypoxic respiratory failure secondary to AVMs. Such a study should have well-defined inclusion criteria, and a protocol for the use of iNO with longitudinal echocardiography to assess causality and response. Furthermore, such a randomized trial may provide a rationale to guide future practice with iNO use in the treatment of PH secondary to AVMs.

## Conclusion

This retrospective matched cohort study concludes that the infants with PH secondary to AVMs (cases) who received treatment with iNO did not respond (oxygenation index) differently compared to babies presented with PH without underlying AVMs (controls) who received treatment with iNO. A large proportion of cases with AVMs has been associated with right ventricular (RV) cardiac dysfunction and mortality. This is significantly higher compared to other causes of PH. Early management of RV dysfunction could be an essential consideration in infants with PH secondary to AVMs compared to infants with other causes of PH.

## Abbreviations

AV: Arteriovenous
AVM: Arteriovenous malformation
BAVMs: Brain arteriovenous malformations
CHF: Congestive heart failure
DAVFs: Dural arteriovenous fistulas
iNO: Inhaled nitric oxide
NICU: Neonatal intensive care unit
PAVFs: Pial arteriovenous fistulas
PBF: Pulmonary blood flow
PH: Pulmonary hypertension
PPHN: Persistent pulmonary hypertension
RV: Right ventricular
SVC: Superior vena cava
TAPSE: Tricuspid annular plane systolic excursion
TnECHO: Targeted neonatal echocardiography
VOGMs: Vein of Galen malformations
